# Raving Rhymes

**Published:** 1854-02

**Authors:** 


RAVING RHYMES.—A patient in one of the insane retreats of England, the subject of a deep melancholy and hypochondriasis, was often heard to say of himself, "I have no soul; I have neither heart, liver nor lungs, nor anything at all in my body, nor a drop of blood in my veins. My bones are all burnt to cinders; I have no brain; and my head is sometimes hard as iron, and sometimes as soft as a pudding."

A fellow-patient, also a hypochondriac, and entertaining as great absurdities himself, drew the following picture of his neighbor:

A miracle, my friends, come view
A man, admit his own words true,
Who lives without a soul:
Nor liver, lungs nor heart has he,
Yet sometimes can as cheerful be,
As if he had the whole.

His head, (take his own words along,)
As hard as iron, yet ere long
Is soft as any jelly;
All burnt his sinews and his lungs;
Of his complaints, not fifty tongues
Could find enough to tell you.

Yet he who paints his likeness here,
Has just as much himself to fear,
He's wrong from top to toe;
Ah, friends! pray help us if you can,
And make us each again a man,
That we from hence may go.

